# Openstage: A Low-Cost Motorized Microscope Stage with Sub-Micron Positioning Accuracy

**DOI:** 10.1371/journal.pone.0088977

**Published:** 2014-02-26

**Authors:** Robert A. A. Campbell, Robert W. Eifert, Glenn C. Turner

**Affiliations:** Cold Spring Harbor Laboratory, Cold Spring Harbor, New York, United States of America; University of Queensland Diamantina Institute, Australia

## Abstract

Recent progress in intracellular calcium sensors and other fluorophores has promoted the widespread adoption of functional optical imaging in the life sciences. Home-built multiphoton microscopes are easy to build, highly customizable, and cost effective. For many imaging applications a 3-axis motorized stage is critical, but commercially available motorization hardware (motorized translators, controller boxes, etc) are often very expensive. Furthermore, the firmware on commercial motor controllers cannot easily be altered and is not usually designed with a microscope stage in mind. Here we describe an open-source motorization solution that is simple to construct, yet far cheaper and more customizable than commercial offerings. The cost of the controller and motorization hardware are under $1000. Hardware costs are kept low by replacing linear actuators with high quality stepper motors. Electronics are assembled from commonly available hobby components, which are easy to work with. Here we describe assembly of the system and quantify the positioning accuracy of all three axes. We obtain positioning repeatability of the order of 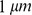 in X/Y and 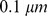 in Z. A hand-held control-pad allows the user to direct stage motion precisely over a wide range of speeds (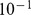 to 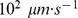), rapidly store and return to different locations, and execute “jumps” of a fixed size. In addition, the system can be controlled from a PC serial port. Our “OpenStage” controller is sufficiently flexible that it could be used to drive other devices, such as micro-manipulators, with minimal modifications.

## Introduction

Two-photon microscopy [1], [2] has gained great popularity in over the last 20 years for both functional [3], [4] and structural [5], [6] studies. The optical path of a two-photon microscope is relatively simple and doesn't have strict alignment tolerances. As a consequence, investigators can easily build their own microscope from commonly available components [7] and control the acquisition with open-source software [8], [9]. A home-built microscope allows researchers to tailor the rig to their specific needs and also provides substantial cost savings.

In addition to the microscope itself, a 3-axis motorized stage is important as it enables a range of applications such as time-lapse imaging of multiple locations in a sample, automated tracking, building image mosaics, and acquiring 3-D (volume) data. However, commercial motorization solutions are expensive and in many cases not designed with microscopy in mind. Whilst cheap linear translator are available, the motorized actuators and the controller units to drive them cost many thousands of dollars. These costs are in addition to the hardware required to build the stage itself. In this paper we present a complete low-cost stage motorization solution comprising a 3-axis drive system and stand-alone controller unit. Our system can be assembled for no more than about $1000, which is about 10 times cheaper than some commercial alternatives. We dub our system “OpenStage” in recognition of the open source Arduino microcontroller at its heart.

The positioning accuracy of our system compares favorably to published specifications of commercial motorized linear stages costing several times as much. The hardware combination used on our rig allows a **minimum incremental motion** of 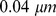 in Z and 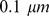 in X/Y. **Unidirectional positioning repeatability** is important in many forms of time-lapse imaging (e.g. Z-stacks) and describes the accuracy with which the stage can return repeatedly to the same position when approached from the same direction. X/Y repeatability is 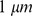 or better. Z repeatability is 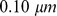 or better. In the more critical Z-axis, we determined that **unidirectional positioning accuracy** was about 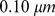. This number describes the accuracy with which a desired absolute position is attained when approached from the same direction. Errors are up to 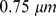 in Z when an absolute position is approached from either direction (**bidirectional positioning accuracy**). However, we found that over longer time periods, accuracy was influenced more by thermal expansion and contraction of the microscope body than by the properties of our drive system. We commonly employ **speeds** of up to 1.2 mm/s in Z and 1.8 mm/s in X/Y. We describe how these specifications can be improved if desired.

Stage motion can be directed either using a hand-held controller (PlayStation 3 DualShock 3 game pad), or programmatically via a PC serial port. The gamepad is a very flexible and intuitive input device. Features include:

1. Fine graded control of speed and direction of all axes using the two analog input sticks.
2. Fixed size motion steps using direction pad (+ pad).
3. User-selectable maximum speed.
4. Storage of up to four different stage positions and return to these positions on the fly.
5. Complete freedom to re-program the functioning of the gamepad by modifying the OpenStage source code.
6. All gamepad buttons return both binary (pressed/not pressed) and analog (how hard it was pressed) values, providing many customization options.

Low cost is not the only advantage of OpenStage. Unlike commercial alternatives, our controller software can easily be modified, greatly increasing its flexibility. For example, it would be possible to modify the unit to provide external control over additional devices, such as Piezo focusing units. The controller could even be adapted for use with other devices, such as micromanipulators or motorized microtomes.

To implement our system the user must have assembled a stage where linear translation can be elicited by rotary motion. We employ micrometers, which provide a low gear ratio, but lead-screw or rack and pinion gears would also work. In this paper and on our website (*turnerlab.cshl.edu/openstage.html*) we make the following available to the community: 1) Parts list and wiring instructions for the required electronic components. These parts are commonly available and easy to assemble without specialist knowledge. 2) Parts list and assembly instructions for the drive hardware. 3) Microcontroller source code to run the controller unit. 4) Criteria for choosing suitable alternative stepper motors and drive hardware. 5) A set of MATLAB and Python functions that interface with our stage controller via a serial port.

## Results

At the outset, our principle design goals were positioning accuracy and cost. We wanted to build a system with adequate positioning accuracy for our experiments (*in vivo* functional imaging in *Drosophila*). We sought absolute positioning accuracy and repeatability better than 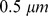 in Z, the most critical axis. In X and Y, repeatability better than 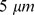 would be adequate for our purposes. Small positioning errors in X and Y can easily be corrected off-line if needed, whereas focusing errors cannot. Given the small size of the fly brain, the system in which we work, a 1 mm/s positioning speed seemed a reasonable goal, since this would allow us to jump from any one region of the brain to another in under about 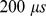 and often much less. We aimed to achieve these specifications using parts costing under $1,000 for 3 axes of motion.

An important secondary design goal was to make it easy for other investigators to modify or enhance our system. To this end, all hardware and software are open-source and our microcontroller code is extensively commented and intended to be understood by someone unfamiliar with both our system and microcontrollers in general. We release our design and source code with the hope that others will use it, improve it, and feed back enhancements to the community. We have set up a website where the latest version of our controller software is available (*turnerlab.cshl.edu/openstage.html*).

### Stage hardware and motorization

**Figure 1:**
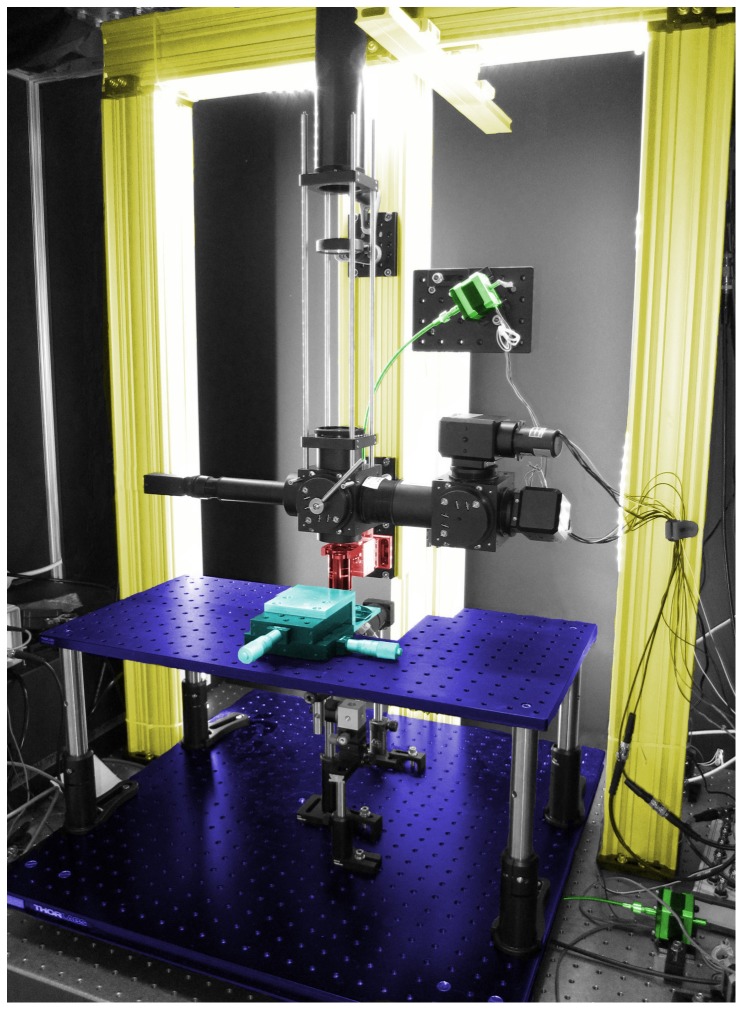
Complete stage setup. This color-coded image shows the main components of our microscope stage. The gantry is constructed out of ThorLabs XT95 rails (yellow). The objective is mounted on a linear translator (red) at the gantry's center. A 24′′ by 24′′ breadboard forms the X/Y stage (1. purple, ThorLabs PBG11105). A raised sub-stage (2. purple, ThorLabs MB1224) brings the specimen up to the level of the objective which is about 13′′ above the surface of the air table. The specimen is mounted on an independently movable platform (cyan), allowing its position to be manipulated manually with respect to the rest of the stage. Our motorization hardware (motors, couplers, and flexible shafts) are colored green.

**Figure 2:**
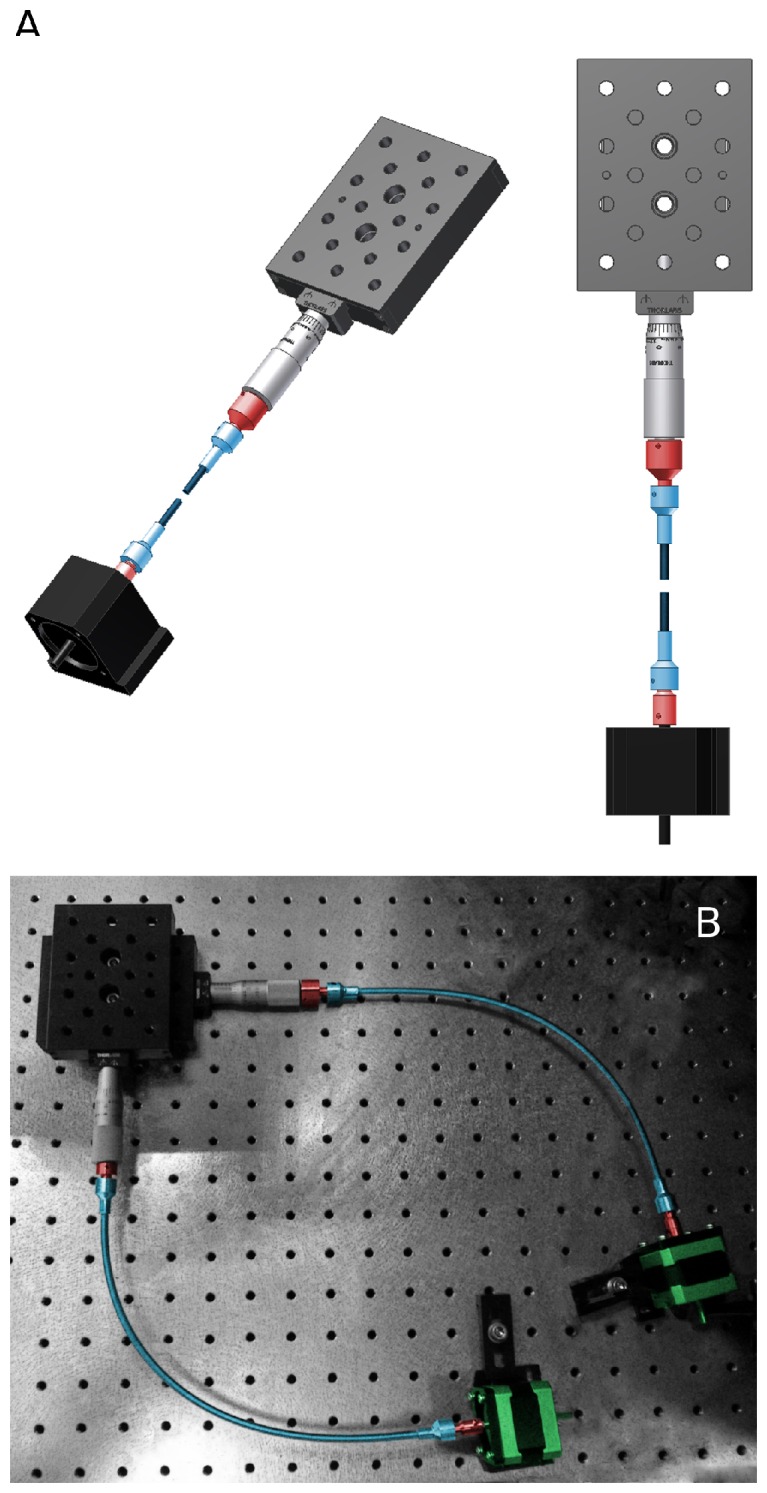
X/Y Drive system hardware. A. Two different views of the X/Y drive system hardware for one axis. A ThorLabs PT1 linear translator with micrometer (top of image) is coupled to a stepper motor via a flexible shaft (colored blue). The flexible shaft (normally curved but here shown “broken”) is attached to the stepper motor shaft and the micrometer thimble via two cylindrical male/female adapters machined in-house (colored red). For scale: holes on the PT1 stage are located 1′′ apart. B. Image of the assembled X/Y translator pair. Two PT1 stages are mounted at right angles to one another and bolted to the air-table. Cylindrical couplers are colored red. Flexible shafts (colored blue) are 12′′ long (SDP/SI. part number A 7C12-12633). Motors (OrientalMotor.com, part number PK243M-02BA) are colored green. Pitch of the holes on the air-table is 1′′. Electrical cables have been removed from this image for clarity.

**Figure 3:**
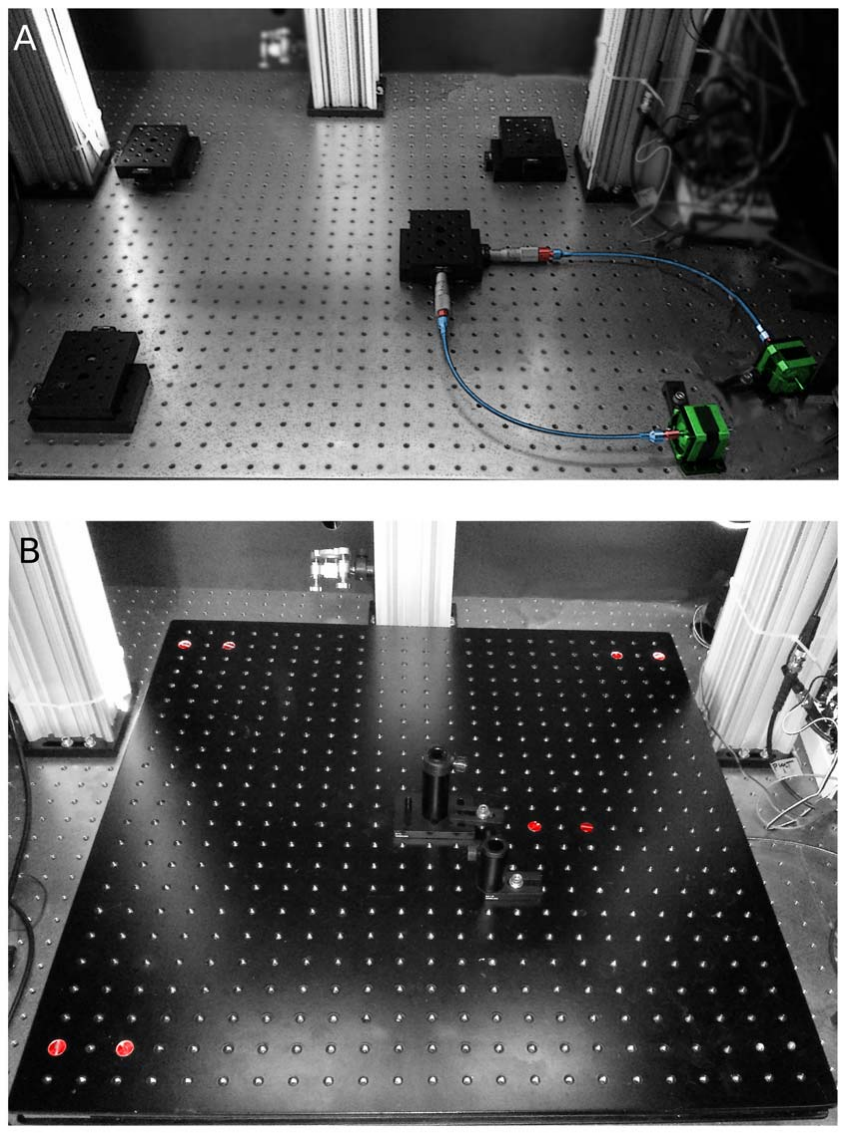
X/Y Stage. A. The 24′′ by 24′′ X/Y stage rests on 4 pairs of PT1 translators. One pair is actuated by micrometers and driven by the motors (colored). The other three pairs have no micrometers and the internal tensioning are removed. Placing the driver translators near the middle of the stage proved essential for obtaining reproducible motions. B. The bread-board bolted to the 4 translator pairs. Screws (colored red) highlight the locations of of the translators.

Our 3-axis stage is integrated into our custom-built 2-photon microscope (Fig.1, where key components are color-coded). The microscope is built around a gantry that places the objective over the middle of a lightweight 24′′×24′′ ThorLabs aluminum breadboard. The objective is mounted to a single linear translator, which allows for focusing. Four pairs of linear translators couple the X/Y stage to the air-table and allow it to be moved with respect to the objective (Figs. 2 & 3). Motion in X, Y, and Z is actuated by micrometers (not visible in Fig. 1) coupled to stepper motors via a flexible shaft. The specimen itself sits on a raised sub-stage to enable easy access to it from a variety of angles (including from underneath). The sub-stage is a 12′′ by 24′′ breadboard raised about 10′′ above the base breadboard using 4 posts. Our specimens are mounted on a small custom-machined platform that can be translated in X and Y by two ThorLabs PT1 linear translators (cyan, Fig.1) located on the raised breadboard. This second pair of translators is optional and allows the sample to be accurately positioned with respect to other components on the rig.

#### Motorizing a translator

Micrometer-actuated linear translators are widely available and reasonably priced, but are designed to be used manually. To motorize them, we used a stepper motor connected to the micrometer via a flexible shaft (Figure 2, see Methods for details). The flexible shaft is required because the micrometer head moves in and out as it rotates. The reason our system is low-cost is partly because we have substituted expensive linear actuators for stepper motors. The CAD drawing in Fig. 2A shows a micrometer-actuated linear translator (Thorlabs, PT1) connected to a generic stepper motor. The motor rotates the micrometer via the flexible shaft (colored blue and shown “broken”). Two cylindrical male/female adapters machined in-house couple the shaft to the motor spindle on one side and the micrometer thimble on the other. These couplers are 1/4′′ male on one side, to fit into the socket on the flexible shaft, with an appropriate sized female connection on the other side, to connect to a micrometer or motor spindle. All connections are secured with set-screws. Note that only the coupler linking the thimble to flexible shaft is completely necessary. The flexible shaft can be attached directly to the smaller diameter motor spindle, doing so merely misaligns the axes of the motor spindle and flexible shaft.

Figure 2B illustrates how the assembled system appears in practice. Here, a pair of PT1 linear translators are bolted to the air table and connected at right angles to provide motion in X and Y. Twelve inch long flexible shafts connect the motors to the micrometers.

#### X/Y-Stage Hardware

Moving the X/Y stage required four pairs of PT1 translators (Fig. 3A). As described above, one pair is actuated by micrometers and driven by stepper motors. One revolution of the micrometer advances the stage by 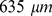. The other three pairs act purely as supports: they have no micrometers and the internal tensioning springs are removed. The assembled breadboard, resting on the four translator pairs, is shown in Fig. 3B. Note that the driver X/Y translators are located near the middle of the stage, close to the center of mass. This turned out to be critical for achieving reproducible motions with this large stage. We initially attached the driver translators at the bottom right corner, but we found this configuration often led to positioning errors of about 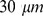 (data not shown). We traced this error to play in the PT1 bearings, which allows the stage to pivot (rotate) about the driver translator pair. Thus, when the stage is driven in one direction, say in X, from a corner, a small rotational movement is also produced. This occurs because the stage is both large (24′′ by 24′′) and heavy (about 17 kg), allowing it to act as a lever. Placing the driver translator pair near the center of gravity of the stage decreases its leverage and eliminates problematic rotational motions.

We also tried supporting the stage using three points of contact: one driven pair of translators and two large ball bearings (not shown). We found this arrangement made it very difficult to achieve orthogonal motions unless the three points of contact were at precisely the same heights. Although it was possible to align the stage and achieve somewhat acceptable motions, alignment was usually lost again after a few days. These problems vanish when the roller bearings are replaced with PT1 translators.

#### Z-Stage Hardware

**Figure 4:**
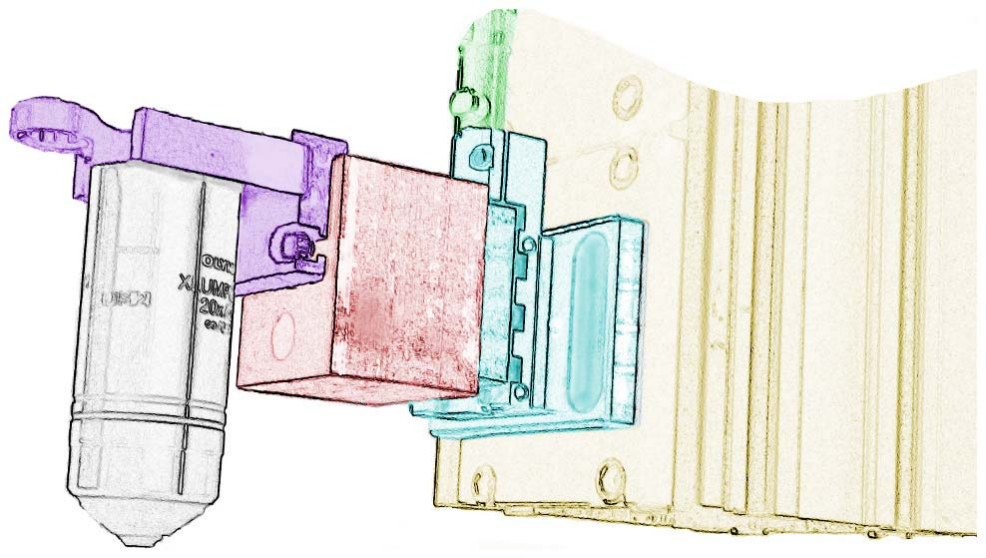
Z Stage. This color-coded drawing shows the assembly of the focusing unit (the region colored red in Fig. 1). The descending XT95 gantry rail is colored yellow. A Newport 461-X-M translator (cyan) is coupled to the rail and actuated by a Newport HR-13 micrometer (green). A custom machined aluminum block (red) couples the translator to a custom machined objective holder (purple). The objective shown (grey) is the Olympus XLUMPlanFLNW 20x used for the measurements in this report.

The objective is suspended over the X/Y stage using a gantry (Fig. 1) built from ThorLabs XT95 optical rails. The colored drawing in Figure 4 shows how the objective is connected to the optical rail via a Newport linear translator. The translator is actuated via a micrometer (partially shown and colored green) that is driven by a stepper motor via a flexible shaft (not shown). One revolution of this micrometer advances the Z-stage by 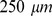. In our microscope, the objective is connected to the translator using parts machined in-house: an aluminum block acts as a spacer and connects the quick-release objective holder to the Z-translation stage.

### Drive system considerations

It is straightforward to connect a stepper motor to a micrometer via a flexible shaft, but some components work better than others so it is important to choose the correct hardware for the task. Flexible shafts are manufactured in various lengths and with various torque ratings. Shafts that are more flexible tend to have lower torque ratings. We tried a range of different shafts and found that those with low torque ratings exhibited substantial backlash (see Methods for details on torque ratings). All three axes are driven by Vexta stepper motors (Table 1). Stepper motors advance by a fixed step size each time a TTL pulse is provided to their driver board. Stage position can be inferred by knowing the step size and the number of steps taken.

**Table 1:** X/Y and Z axes motorization components.

Item	Supplier	Part name (X/Y)	Part name (Z)
Stepper motor	OrientalMotor.com	PK243M-02BA	PK243M-01BA
Mounting clamp	Oriental Motor.com	PAL0PA	PAL0PA
Flexible shaft	sdp-si.com	A 7C12-12633 ( 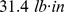 )	A 7C12-10633 ( 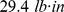 )

Components used to motorize the X, Y, and Z linear translators of our stage. The Z-stage components provide for higher speeds and there is no reason why these could not be used on all stage axes. At the time of writing the above component costs are $115 per axis. Couplers to connect these parts were machined in-house (Fig. 2) and cost $83 per axis. This cost can be halved by using only one coupler (see Methods), or eliminated completely if no couplers are used or if the user has machining experience.

The **resolution** and **speed** of the drive system are affected by the choice of stepper motor. **Resolution** is affected by both step size and torque. Steppers can be purchased in a variety of step sizes, with 1.8° and 0.9° being common. A full step of a 0.9° stepper motor will advance a stage by 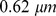 if it is coupled to a 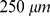/revolution micrometer (Table 2). So called “micro-stepping” can increase resolution, by allowing the motor to advance by fractions of a full step. Micro-stepping is conducted by the stepper motor driver board and common fractional step sizes are 1/2, 1/4, 1/8, and 1/16 steps. Whilst, say, sixteen 1/16 steps always advance the motor by one full step, the sizes of the individual micro-steps are not identical and vary across the stepping cycle. The “linearity” of the stepper motor describes the homogeneity of the micro-step sizes across the stepping cycle. Micro-stepping cannot be used to obtain infinitely fine resolution since torque decreases with micro-step size. For example, 1/16 steps exert only 9.8% of the full holding torque. Thus, if the net load on the motor is greater than the torque exerted by a single microstep, then multiple microsteps may need to be taken before the motor will move. For this reason, a motor with higher torque may provide higher resolution. The maximum **speed** of a stepper motor is influenced by various design factors. One important point, however, is that a motor's torque decreases as speed increases and some motors with a high holding (peak) torque may be unable to produce high speeds. Thus it is important to consider the whole speed/torque curve when selecting a suitable motor. Motors driven beyond their limits will skip steps or stall, which is not acceptable for an open-loop system such as ours, where motor position is not verified using encoders. However, when operated well within their rated speed and torque ranges, stepper motors are very reliable.

**Table 2:** Step size and speed for common stepper/micrometer combinations.

Full Step	635 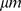 /rev.	250 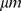 /rev.	125 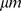 /rev.
1.80°	3.18, 1.59, 0.20 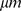	1.25, 0.62, 0.08 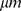	0.62, 0.31, 0.04 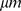
	12.70, 6.35, 0.79 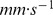	5.00, 2.50, 0.31 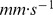	2.50, 1.25, 0.16 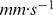
0.90°	**1.59, 0.79, 0.10** 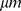	**0.62, 0.31, 0.04** 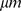	0.31, 0.16, 0.02 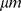
	**6.35, 3.18, 0.40** 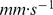	**2.50, 1.25, 0.16** 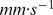	1.25, 0.62, 0.08 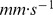
0.72°	1.27, 0.64, 0.08 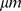	0.50, 0.25, 0.03 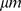	0.25, 0.12, 0.02 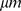
	5.08, 2.54, 0.32 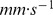	2.00, 1.00, 0.12 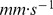	1.00, 0.50, 0.06 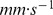
0.36°	0.64, 0.32, 0.04 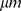	0.25, 0.12, 0.02 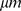	0.12, 0.06, 0.01 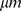
	2.54, 1.27, 0.16 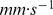	1.00, 0.50, 0.06 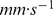	0.50, 0.25, 0.03 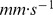

The step sizes and maximum speeds available with a range of stepper motors and micrometer gear ratios. Three micrometer gear ratios are ranged along the columns. ThorLabs PT1 imperial micrometers are 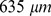/rev (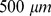/rev are also available). The Newport HR-13 provides 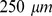/rev and the bulky Newport HR-1 provides 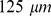/rev. Stepper motor full step sizes are ranged along the rows. Here we list all 4 step sizes available from by Vexta (OrientalMotor.com). Each table cell lists the step sizes and theoretical maximum speeds that can be attained for a given motor/micrometer combination. The top row of each cell lists step sizes for full steps, half steps, and 1/16 steps. The bottom row of each cell lists the corresponding theoretical maximum speeds. Maximum speed assumes our OpenStage microcontroller code running at a maximum step pulse rate of 4.0 kHz (in principle this can be increased). Bold entries correspond to the combinations described in this paper.

The stepper motors we chose have a full step size of 0.9° (400 steps per rev.) and a holding torque of about 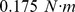 (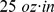). Informal observation suggests that a stepper motor driving the stage hardware will stall at RPMs corresponding to a torque of about 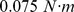 (see Methods for details). We drove the motors with a “Quadstepper” driver board (see Methods), which is capable of executing micro-steps as fine as 1/16 of a full step, yielding a minimum stage motion of about 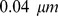 in Z and 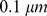 in X and Y.

Our approach is to utilize stepper motors in open-loop but to characterize the units sufficiently to ensure they are being driven within their operating range and so do not skip steps. As we will show, when used correctly an open-loop stepper motor solution is excellent for controlling a 2-photon microscope stage. We found that closed loop control, which is more expensive and harder to implement, was not necessary to achieve reliable positioning. Using stepper motors in this fashion is not unprecedented, and is commonly implemented in hobby CNC machines (see *github.com/grbl/grbl*).

### Controller features

Our motor controller is built around an Arduino Mega 2560, which is based on the Atmel ATmega1280 microcontroller. A microcontroller is a single integrated circuit containing a processor, memory, and multiple input and output lines. The Arduino is programmed in C or C++ and ships with a beginner-friendly development environment that, among other things, simplifies the process of compiling code and uploading it to the ATmega chip.

**Figure 5:**
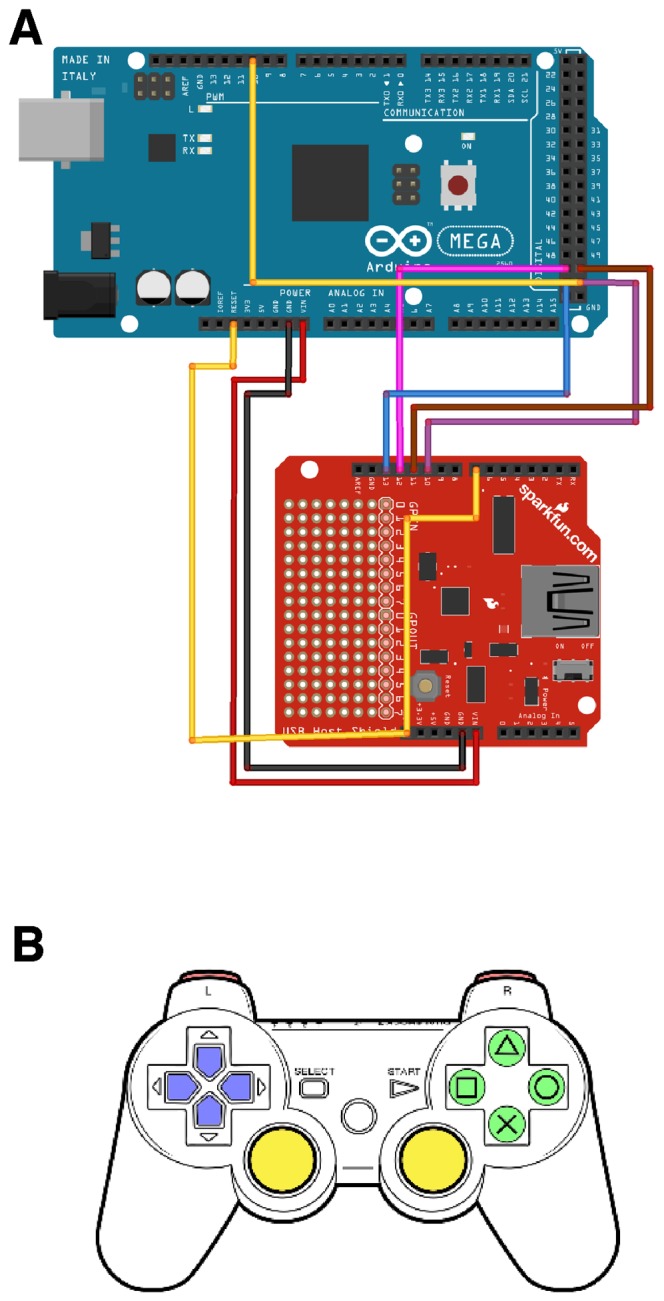
USB interface and DualShock layout. The DualShock has a mini-USB socket which allows it to be connected to the Arduino Mega via an interface board known as a USB Host Shield. A. Wiring diagram for connecting a USB Host Shield to an Arduino Mega 2560. We include this here as instructions on-line are hard to find. B. The PlayStation3 DualShock controller. The buttons currently used by OpenStage are colored. Red: shoulder buttons (L1 and R1) select the Speed Mode. Blue: direction pad, executes fixed-step motions in X, Y or Z (for Z the user holds down the Triangle button whilst pushing up or down). Yellow: analog sticks provide motion in X, Y, (left stick) and Z (right stick). Green: right-hand buttons (Triangle, Circle, Cross, and Square) allow the user to store and go to four different set point locations.

OpenStage provides all the basic features of a commercial controller. Stage position and speed are reported on an LCD display. A serial connection allows the stage to be controlled programatically by a PC, or the user may issue commands manually with a PlayStation3 DualShock controller. The DualShock is connected to the Arduino via a USB Host Shield (see Fig. 5A and Methods for details) and is an excellent interface device for controlling a microscope stage. The DualShock controller provides the following functionality:

- *Analog control* of speed and direction for each axis is provided by the two analog sticks (Fig. 5B, yellow). The left stick controls X and Y and the right stick controls Z. Larger stick displacements are mapped to higher motion speeds, making it easy to manually guide the stage at exactly the desired speed.
- *Fixed-size motion steps* are provided by the direction pad (D-pad, Fig. 5B, blue). For instance, pressing the left D-pad button moves the stage one increment to the left. By default the D-pad controls X/Y motions; holding down the Triangle button whilst pressing up or down on the D-pad yields fixed step size motions in Z.
- The two shoulder buttons (L1 and R1, colored red in Fig. 5B) allow the user to cycle between 4 different *Speed Modes*. The selected speed mode influences both the maximum speed of analog stick motions and the size of the D-pad motion steps. Speed mode #1 provides a nominal maximum speed of 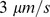, #2 is 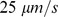, #3 is 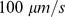, and #4 is 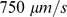. The size of the D-pad steps range from 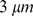 to 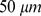. Changing the speeds and motion steps sizes involves only trivial modifications to the source code. The currently selected Speed Mode is indicated by the 4 LEDs on the DualShock.
- The user can *store* up to 4 locations and return to them later using the 4 right-hand buttons (Fig. 5B, green). The user presses firmly on a button for 1 second to store the current stage position. The controller produces an audible indication when a new location is stored. The user can return at any time to the stored location by double-clicking on the corresponding button. OpenStage returns to the stored position at maximum speed.

Finally, OpenStage accepts serial commands which provide control over most aspects of the stage (see Tables 3 and 4). We provide a set of example MATLAB and Python functions that use this serial protocol to interface with the stage. To minimize the risk of skipped steps, all motions that do not go through the analog sticks are subject to smooth accelerations and decelerations. We implemented this using the AccelStepper library (www.airspayce.com/mikem/arduino/AccelStepper). More detailed information on the serial protocol, electronics, and wiring can be found in the Methods and in the controller source code.

**Table 3:** Serial control interface: Go To motions.

Command	Command sent from PC	Data received	Details
Go To Absolute Position	‘ga’ X,Y,Z‘$’	‘$’	Sends ‘ga’ followed by comma separated integers defining the target for each axis. Numbers may be signed. Three least significant digits denote digits after the decimal point. Motion completion is signaled from the stage with a terminator character.
	e.g. to move 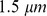 in Z: ga0,0,1500$		
Go To Relative Position	‘gr’ X,Y,Z ‘$’	‘$’	As ga, but executes relative motion.
Set Step Size	‘ss’ n	none	Set stepper motor step size for right-button seek motions and serial Go To motions. n is an integer between 1 and 5, mapping onto full, 1/2, 1/4, 1/8, and 1/16 steps respectively.
Receive Step Size	‘sr’	n ‘$’	Report step size for right-button and Go To motions. Step size reported as a float defining the fraction of a full step the stage takes. All axes share the same step size.
Set Velocity	‘vs’ X,Y,Z ‘$’ e.g. to set 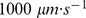 on all axes: vs1000,1000,1000$	none	Must provide as many values as there are axes.
Read Velocity	‘vr’	X,Y,Z ‘$’	Returns three integers defining maximum velocity for right-button and Go To motions on each axis.
Set Acceleration	‘as’ X,Y,Z‘$’ e.g. to set 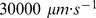 on all axes: as30000,30000,30000$	none	Must provide as many values as there are axes.
Read Acceleration	‘ar’	X,Y,Z ‘$’	Returns three integers defining the acceleration for right-button and Go To motions on each axis.

Commands for initiating and controlling stage motions via the serial port. Speed, step, and acceleration settings also affect right-hand (stored location) motions initiated by the DualShock. Command descriptions in this table assume a 3-axis stage. If the stage comprises a different number of axes then the number of transmitted values will be adjusted accordingly. i.e. “Empty” values will are not be sent. Default baud rate is 115200. The terminator character is ‘$’.

**Table 4:** Serial control interface: misc. commands.

Command	Command sent from PC	Data received	Details
Tell Position	‘p’	X,Y,Z‘$’	Returns comma separated floats in ASCII terminated with $
Zero Stage	‘z’	none	Zero stage position counters. Zeros values on LCD display and values returned by Tell Position command. Stage retains the absolute position of any right-button stored set points. No terminator need be sent.
Set Speed Mode	‘m’ n	none	Sets the Speed Mode used for interactive motions on the DualShock. Illuminated LED on DualShock changes accordingly. n is an integer between 1 and 4.
Show Information	‘I’	ASCII text	Sends a collection of information about the stage to the PC. Information includes axes gear ratios, step sizes, and speeds, accelerations, and RPM of Go To motions.
Beep	‘b’	none	Issues a short beep from buzzer on controller.

Commands for obtaining information from the stage or controlling other features, not related to automated movements. Command descriptions in this table assume a 3-axis stage. If the stage comprises a different number of axes then the number of transmitted values will be adjusted accordingly. i.e. “Empty” values will are not be sent. Default baud rate is 115200. The terminator character is ‘$’.

### Positioning repeatability in X and Y

Our drive system allows us to translate the entire stage in X and Y, and to focus by moving the objective in Z. We will begin by quantifying positioning repeatability in X and Y. We imaged pollen grains spread out on a slide and measured the accuracy with which we could repeatedly center the objective over three different parts of the slide. We chose three pollen grains located about 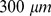 to 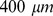 apart and used serial port commands to cycle between them repeatedly 50 times. Upon arriving at each pollen grain, we acquired a single 512 by 512 frame at an optical zoom factor of 2 (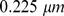 per pixel). Positioning error across the 50 cycles was measured using a sub-pixel image registration algorithm [10], which we have previously [11] used for motion correcting *in vivo* functional recordings. We can use this algorithm to calculate the number of pixels (and hence microns) by which subsequent frames are displaced from the first frame. A displacement of zero would indicate that the stage returned exactly to the starting position.

**Figure 6:**
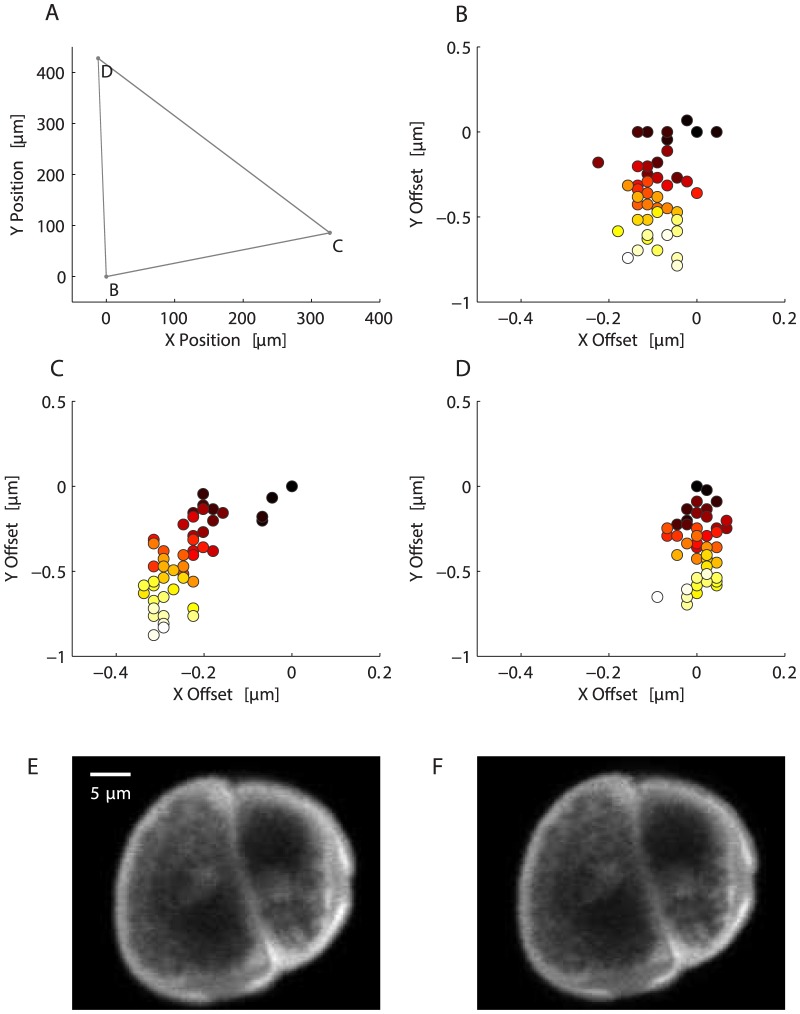
X/Y positioning accuracy. A. The X/Y stage was moved 50 times between three different pollen grains on a sample slide. The sampled pollen grains were about 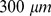 to 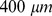 apart. The points show the final position of the stage and the grey lines link these points, indicating the path taken by stage. At each of the three points (labeled B, C, and D in reference to the sub-plots with which they are associated) there are 50 data points, which at this scale can not be resolved. B-D. The positioning errors at each pollen grain. The colors indicate cycle number, with black being the first observation and white the 50th observation. The distribution of the colors indicates that the positioning errors are not random, and the stage drifts about 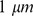 over these 50 positioning cycles. E. Close-up image of the pollen grain at position C. This image is obtained by averaging the raw frames and so it is slightly blurred due to the positioning errors. F. Same as E, but frames were aligned before averaging and so slightly more detail is visible.

Figure 6A shows the path taken by the stage. The three vertices are the locations of the pollen grains. Positioning errors are very small and so at this scale the 50 data points at each vertex can not be resolved. Figures 6B–D show X/Y positioning errors for each pollen grain. The color scale corresponds to the motion cycle number, and so indicates the order in which the data were acquired. The first data point is black and the last is white. The colors are not distributed randomly, which indicates that there is systematic drift. The magnitude of this drift is small, however, being no more than about a micron over these 50 motion cycles. In order to put this level of drift into context, Fig. 6E shows an image of pollen grain C. This image was obtained by averaging the 50 raw frames, meaning that the X/Y positioning errors are free to add blur to the image. Figure 6F is an image of the same pollen grain, but here the mean is constructed from the motion-corrected frames and so there is no added blur due to positioning errors. The differences between the two images are subtle, with the motion-corrected version being only slightly more detailed.

High accuracy in Z is usually much more important than high accuracy in X and Y. This is because X/Y positioning errors can usually be corrected off-line with image registration techniques, whereas positioning errors in Z cause the sample to be out of focus and so can not be recovered. Thus, in the remainder of the paper, we concentrate on quantifying positioning accuracy in Z.

### Quantifying Z motion

**Figure 7:**
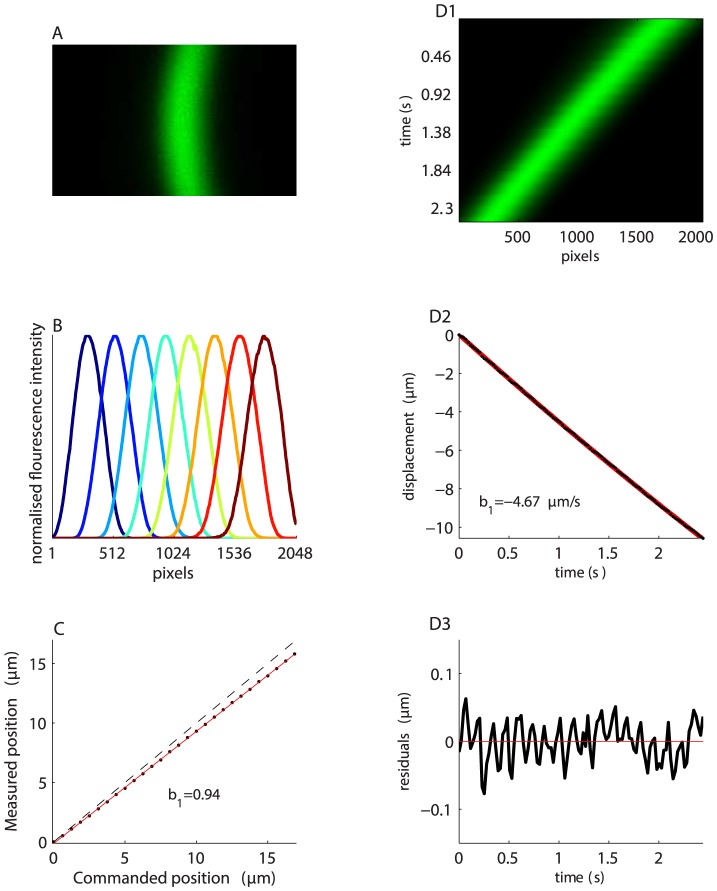
Validation of Z-positioner. A. Single frame obtained by imaging a fluorescent slide which has been tilted by raising it on one side. The slide's tilt leads to a fluorescent vertical bar, since regions of the slide to the right and left of the bar are out of focus. B. Cross section of the bar's position as the objective is advanced in 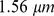 steps. C. Measured position of the Z-stage as a function of commanded position as the stepper motor is advanced in full steps (0.9°, which should result in motions of 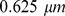). The slope of the regression line (red) is almost 1.0 (dashed line), indicating that the stage moves close to the commanded values. D1. Smooth motion of the bar over time under the control of the DualShock gamepad's analog input stick. Each row corresponds to a different frame. D2. Displacement of the objective as a function of time. Linear regression in red. D3. Residuals of regression from D2. The small periodic errors are due to non-linearities across the stepper motor's micro-stepping cycle.

Evaluating positioning accuracy in Z is less straightforward than X and Y since objective motion alters focus. To measure the performance of our z-stage we used the “tilted slide trick”, which converts changes in focus into an apparent X translation of the specimen. We coated a glass slide using a green fluorescent marker pen, and sealed the marking with a coverslip. This creates a very thin fluorescent layer which we can excite using 920 nm laser light and collect emitted green fluorescence using the same detection path we would normally use for GFP. The slide was placed on the microscope stage and elevated on one side by 5.5 mm using a nut (see Methods for details). The fluorescent layer was then imaged with an Olympus 20x (XLUMPlanFLNW, NA 1.0) water immersion objective, that produces a field of view of 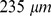 with ScanImage [9] at unity zoom. Due to the tilt of the slide, the fluorescent layer appears as a vertical bar (Fig. 7A). The bar is curved because the slide is not completely flat. Figure 7B shows a series of cross-sections of the bar as the stepper motor is advanced in Z. Since illumination drops off at the field edges, each cross-section was normalized by its maximum value.

In Fig. 7B the bar's location is represented in pixels along the X axis. We can use simple geometry to convert these values to microns. Since we know the 5.5 mm high nut is located 65 mm from the slide's edge (all distances measured carefully with digital calipers), the slide's angle of tilt must be 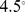. We measured the field of view as being 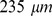 across, and so determined that the slide rises 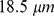 in Z over this distance. Since the image is 2048 pixels across, a bar motion of one pixel corresponds to a Z-stage translation of 9.0 nm. This is an order of magnitude smaller than the Z-stage's theoretical minimum motion step (0.04 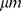, which is the distance the average 1/16 step pushes the Z-stage when coupled to the 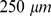/revolution micrometer).

Our first goal was to confirm the validity of the 9.0 nm/pixel calibration factor. We did this by moving the objective in fixed step sizes and comparing the expected objective position (inferred by motor step size and micrometer gear ratio) with the position calculated using the tilted slide. We commanded the objective to advance in single full steps, each of which should move the objective by 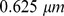 (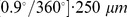). Under these circumstances the stepper motor exerts the highest torque possible and missed steps are very unlikely indeed. Following each step we acquire a frame and recorded the bar position as shown in Figs. 7A & B. The number of pixels by which the bar is displaced in X was calculated using the sub-pixel image registration algorithm.

Figure 7C shows the relationship between commanded and calculated Z position when advancing the objective in 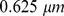 steps. A linear regression (red) yields a slope of 0.94, close to 1.0 (dashed line), which would indicate a perfect correspondence between the observed and theoretical position of the bar. We cannot be certain where the 6% slope error originates since we do not have an external validation of objective position. However, it is probably due to inaccuracies in measuring the tilt of the slide with respect to the objective. Errors in step size are most likely to affect the scatter around the regression line (which is very low) rather than the slope. The micrometer gear ratio being different from the published 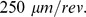 would be consistent with what we observe, but an error of 6% is implausible (Newport Corp. estimates the gear ratio is accurate to better than 0.001%, personal communication). It is reasonable to conclude that the 9.0 nm/pixel conversion factor is accurate to within about 6% and that the error arises mostly from small imprecisions in our measurement of slide tilt. Thus, all measurements of Z position that follow likely contain an error of about 6%. Nonetheless, the tilted-slide clearly allows us to measure Z-stage motions far smaller than the axial resolution of the objective.

### Measuring step linearity

As discussed above, stepper motors are most accurate when advancing in full step increments (e.g. Fig. 7C). Here we test how smoothly and accurately the Z-stage advances when commanded to move at a constant speed in 1/16th micro-steps. We scanned the test slide whilst commanding the objective to travel at 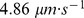. We acquired full frames (31 rows, 2048 pixels per row) at a rate of 43 frames per second. The motion of the bar across the tilted slide is shown by the image in Fig. 7D1, where each row shows the average bar position from one frame. The displacement of the bar is plotted as a function of time (black points, Fig. 7D2). The data were fitted with linear regression (red line, Fig. 7D2) and the slope of the line confirms that the Z-stage is moving at the commanded speed. Figure 7D3 are the residuals of the fit. The periodic nature of the deviation around the fit (red line) is due to non-linearities in micro-step size. The RMS discrepancy between the true position of the Z-stage and the desired position is only 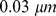 and never more than 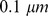.

To demonstrate motion smoothness using a more conventional 3-D sample, we imaged a pollen grain whilst slowly focusing through it using the DualShock controller's analog stick (see Movie S1). The pollen grain in this movie is roughly 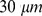 across. The frame rate of both the movie and the acquisition is 7 FPS. The movie shows Z focusing speeds between about 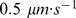 and 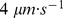. As mentioned above, the controller has the ability to store set-point locations via the right-hand buttons. In Movie S2 a depth near the middle of the pollen grain is stored as the set point. The objective is moved away from this location fairly slowly using the analog stick. The set point is returned to rapidly by double-clicking on the right-hand button which was bound with that depth. Several outward and return motions are shown in the movie.

### Z-stacks

One of the most critical tasks for a motorized focusing mechanism is the ability to perform repeatable Z-stacks. A Z-stack is defined as the process of cycling the objective through a series of pre-defined depths. In some cases, such as functional imaging, it may be necessary to do this cyclically; hitting the same depths multiple times with high repeatability. If this process is not accurate, data loss will result due to focusing errors. We tested the accuracy of our stage by commanding it to acquire 100 identical Z-stacks, each composed of 10 depths spaced 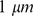 apart. The stacks were acquired in two batches of 50 cycles, with a 10 minute break between the two acquisition periods. We imaged the tilted fluorescent test slide in order to obtain an accurate measure of the objective position. The motions themselves were conducted in half steps at a speed of 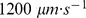.

**Figure 8:**
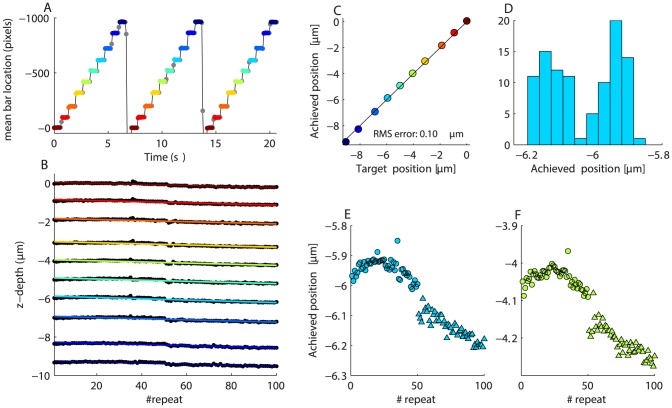
Z-Stack behavior. A. Position of the fluorescent bar as the objective is commanded to upwards in ten steps of 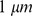. Three cycles of motion are shown. Each point represents data from a single frame. Grey points indicate frames when the Z-stage is moving. Colored points indicate frames when the Z-stage is stationary. Points at the same depth share the same color. Upward motion is indicated by more negative numbers. Positioning accuracy is unidirectional, since the objective always approaches each depth from the same direction. B. The location of the objective over 100 Z-stack cycles. Each point represents objective position from one cycle of one depth. There are 100 points for each depth. Motions are highly repeatable over time. Colored lines are linear regression fits. C. Correspondence between target and achieved position. D. Achieved position for the 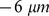 Z-depth. Note the data are bimodally distributed. E. Same data as D, but plotted as a function of stimulus repeat. Different symbols distinguish between data obtained in the first and second blocks (10 minute gap between blocks). F. Same as D, but for the 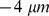 depth.

Figure 8A shows the location of the fluorescent bar's peak over three cycles of the Z-stack. Each point represents depth position from a single frame. Frames were acquired at 12 FPS and not synchronized to objective motion. Individual steps within each cycle were always approached from the same direction, so these data showcase the unidirectional positioning accuracy of the objective. In this case, the Z-stack was obtained from bottom to top, so at the end of each cycle the objective was re-positioned to a location 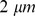 below the first Z depth. We determined the time periods over which the bar was stationary (stationary epochs at the same depth are colored identically) and extracted the mean position at each step. This allowed us to plot positioning repeatability within each depth (Fig. 8B). We fitted linear regressions to each depth (color codes the same as in Fig. 8A). The slope of the lines indicated drift rates of between 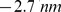 and 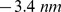 per Z-stack cycle. We plotted the 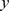 intercepts of the fits as a function of commanded Z position (Fig. 8C). The points are scattered tightly around the blue unity line, indicating a good match between desired and achieved position (RMS of the difference between two is 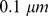).

Whilst the absolute RMS positioning accuracy is good, the small quantity of drift visible in Fig. 8B is a potential concern. Is this drift due to cumulative positioning errors or is it ongoing and not directly related motion of the Z-stage? To explore this issue, we plot a histogram of Z-stage position for the 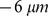 data (Fig. 8D). The data are bimodally distributed. Figure 8E shows Z depth as a function of repeat number for the 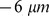 data. The circle and triangle symbols distinguish between the first and second blocks of data (which were recorded with a 10 minute time gap). There's an obvious discontinuity between these data blocks, indicating there was drift even during the 10 minute time period when the objective was stationary. Figure. 8F shows equivalent data from a different Z depth. Thus, the slow drift we see in these recordings appears to be ongoing and therefore not due to missed steps.

We imaged a pollen grain to show what this degree of repeatability looks like in practice with a conventional 3-D sample. In this case, we imaged the pollen grain at two depths separated by 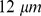. We cycled back and forth between these depths 100 times, obtaining 100 images at each depth location. We then aggregated frames from each depth and display these as a movie (Movie S3) which plays at 20 FPS. There is little or no visual indication of Z drift over these 100 repetitions.

### Z-Stage stability and sources of drift

Figure 8 shows that the Z-stage has good unidirectional positioning accuracy in the context of a Z-stack. The dominant source of error seems to be ongoing drift, because over short periods of time positioning accuracy is well under 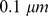 (Figs. 8E & F). What is the source of this drift?

The observed drift is too gradual to be due to missed steps and is more likely related to factors such as shifting of the tilted test slide or thermal expansion of the gantry due to changes in ambient temperature. The drift persists even when one edge of the slide is secured with dental wax (data not shown). We therefore considered the possibility of thermal effects. We calculated that a 1°C increase in temperature would raise the objective with respect to the specimen by approximately 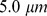, due to expansion of the aluminum gantry. This number takes into account expansion of the X/Y stage support pillars (Fig. 1), which are unable to keep pace with the gantry since they are made of steel which has a linear thermal expansion coefficient that is 70% smaller than that of aluminum. The 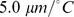 figure is based upon the effective height of the objective, not the full height of the gantry (which is not relevant). Thus, a temperature change of only 0.02°C would result in a shift in objective position of 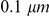, which is of the order observed in Fig. 8.

**Figure 9:**
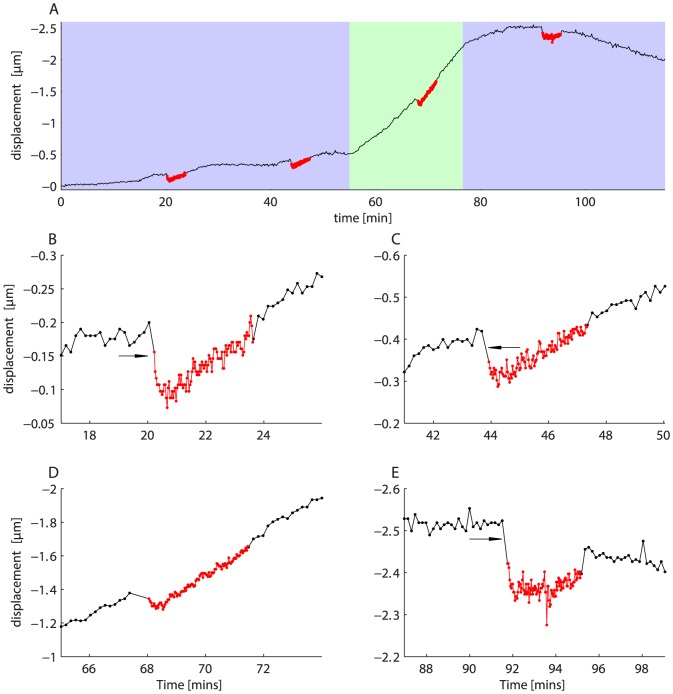
Z-stage drift and absolute positioning accuracy. A. Z-stage drift over a two hour time period (black trace). Each red point represents the return position after one down/up cycle, during which the objective is lowered 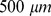 then returned to the initial position. Blue background indicates time periods over which the room air conditioning is active. Green background indicates time period over which the air conditioning is deactivated, and the room warms. Upward motion is indicated by more negative numbers so, as expected, the objective rises when the room warms. Most of the gradual drift in objective position seems to be due to factors not directly related to motion of the drive system. B–E. Detail showing the motion epochs. Red indicates period during which objective motions are being executed. In three cases (arrowed) the objective undershoots on its return by about 0.05 to 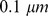 but rate of drift does not alter during the motion epoch. Thus, these larger amplitude motions are conducted with an accuracy similar to that of the 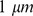 Z-stack motions (Fig. 8).

One possibility is that repeated motion of the objective (e.g. as part of a Z-stack) heats the hardware and leads to expansion. A second possibility is that the thermal expansion is ongoing and due to ambient temperature changes. To evaluate these two possibilities we imaged Z-stage position over a 2 hour period (Fig. 9A). During this recording period we tested the following:

1. Whether ambient temperature affects Z-stage position. We did this by deactivating the room's air conditioning for a 20 minute period in the middle of the recording session. Air conditioner state is shown by the colored background in Fig. 9A (green indicates room air conditioner is disabled). The Z-stage position is measured every 10 seconds whilst the Z-stage is stationary (black points).
2. Whether prolonged Z-motion alters the rate of Z-stage drift or causes Z-stage drift. We did this by moving the objective rapidly down and then back up 100 times. Motion amplitude is 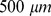 and speed 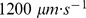. It takes about 2 minutes to complete this motion sequence and we repeated it 4 times during the 2 hour recording period. Motion epochs are shown in red in Fig. 9.
3. We measured unidirectional return accuracy of the objective following each motion by imaging the fluorescent bar position (Figs. 9B–D). This showcases unidirectional positioning accuracy for motions of larger amplitude than those from the Z-stack data shown above.

Figure 9A shows Z-stage drift over the entire two hour period. The objective drifts upwards (more negative numbers) for the first 90 minutes, and drift rate is dramatically increased when the room air conditioner is switched off (green shaded background) causing the room to warm. Reactivating the air conditioner causes the drift rate to slow and eventually reverse direction, as temperature decreases and the gantry contracts. Switching off the air conditioner for 20 minutes caused the objective to rise with respect to the specimen by a height of about 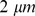. This is consistent with an increase in temperature of approximately 0.4°C. Our microscope is sensitive to the building's cooling system because it is located near the cold air vents and two sides of the light cage are composed of fabric, allowing air to flow. The microscope itself is also designed in a way that makes it prone to drift caused by thermal expansion: the objective is relatively high above the air table surface (13′′), which causes it to move more per unit temperature increase than would a shorter microscope. Furthermore, the steel posts supporting the sub-stage expand at a lower rate than the aluminum gantry.

In comparison with the background drift of the objective height, the commanded unidirectional motions result in minimal absolute positioning error. Errors are of a similar magnitude to those observed during the unidirectional Z-stack. During the first 55 minutes, the objective drifts upwards by only 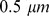. There are two epochs of commanded motion during this time period (Fig. 9B & C). Each red point in these figures shows the *return position* of the objective. i.e. where it ended up, having gone out 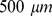 and then back again. Only the return position can be measured because the depth of field of the tilted slide is is under 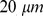. Following the first out and back motion, there is an initial rise in objective height (arrowed) of as much as 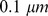 (Fig. 9E). This corresponds to the objective undershooting (failing to return to its starting position) suggesting the drive system has a backlash of roughly 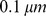 (see next section for details). Within a motion epoch (red), the objective shows very good return reliability (disregarding the drift, RMS deviation around the mean return position is 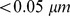 in Figs 9B, C, & E). Taken together, these data show that the objective is able to make 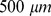 motions with very high absolute and relative accuracy. Motions of this sort are important for tasks such as time-lapse imaging.

### Backlash: measurement and correction

Backlash is a form of hysteresis which originates from the relative movement between components of a drive system. Backlash occurs following a change in drive direction and generally manifests itself as a hesitation in motion initiation. Backlash affects a property of the drive system known as ‘bidirectional repeatability’, which describes the ability of the drive system to achieve a commanded position when approached from either direction. Backlash is an inherent property of geared systems. Whilst the only gear in our system is the micrometer, the properties of the flexible shaft are such that it might contribute significantly to backlash. Following a change in direction, the shaft must be turned by a certain angle whilst it off-loads tension from the previous motion direction and then accumulates sufficient tension to begin rotating the micrometer in the new direction (we have no data on how large this angle might be). Note that a similar effect can also occur in the stepper motor itself: the force required to hold a load is stored in the motor's magnetic field and it can take more than one micro-step to reduce this force and change its direction. If the motor is being driven in half or full steps, this effect is unlikely to occur.

**Figure 10:**
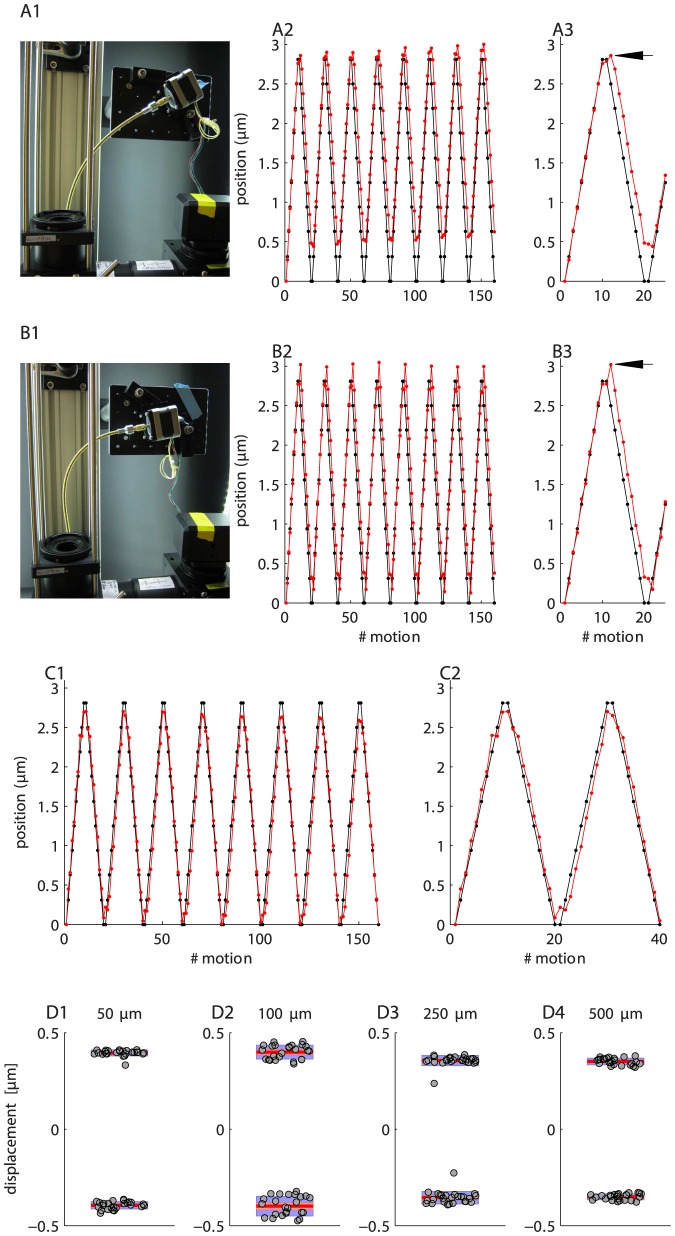
Backlash and bidirectional repeatability. A1. Configuration of the shaft for the data shown in A2 and A3. A2. Desired (black) and achieved (red) objective positions. Each point represented one half step. A3. Detail from A2. Backlash spike is arrowed. B1 to B3. Same as A1 to A3 but for a different shaft configuration. C1 and C2. Same as B2 and B3 but with backlash correction implemented. D1 to D4. Bidirectional repeatability for larger amplitude motions (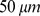 to 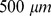). Each panel shows the return position of the focuser. Data are divided according to the direction from which the focuser approached zero. Each data point is a different return cycle. The bars show mean (red line), 95% confidence interval for the mean (pink area), and 1 standard deviation (blue area). All points being at zero would indicate perfect performance.

Since potential backlash in a flexible shaft is related to tension on that shaft, we measured backlash with the shaft configured in two different ways (Figure 10A1 and B1). In Figure 10A1 tension on the shaft is fairly low, whereas in Figure 10B1 it is rather high. This shaft has a nominal radius of curvature of 12′′. The objective was commanded to move up and down in individual 1/2 steps over a distance spanning about 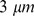. Following each step, the objective depth was assessed using the tilted slide. The results for the first shaft configuration are shown in Fig. 10A2. Each point shows one half step of motion. The black is the desired location and the red is the achieved location. The backlash is highlighted in Fig. 10A3, where it is visible as upward “blip” (arrowed). Thus, in the first step following a direction change, the Z-stage continues to move in the *original* direction and takes one 1/2 step to catch up. This is somewhat unusual, since in a conventional geared system backlash manifests as an initial absence of motion following a direction change. What we see here is likely due to tension in the shaft (or even the motor) slowly releasing. We confirmed this effect is not due to the controller issuing a step command in the wrong direction, by visualizing the step and direction signals on an oscilloscope. A skipped step would not explain what we observe here.

With the shaft at higher tension (Fig. 10B1 to B3) the backlash spikes (arrowed) are slightly larger. Note that the blue tape in Fig. 10B1 is a reference marker that shows the location of the rear of the stepper motor in Fig. 10A1. Despite the fact that the shaft configuration has changed substantially, the motions themselves are relatively unaffected. This is encouraging as it indicates that shaft positioning is not critical.

In order to confirm that the spikes are backlash, we introduced a simple backlash correction procedure. Following a direction change, the stepper was commanded to issue an extra step in the new direction. The results of doing this are shown in Fig. 10C1 & C2. The extra steps are not plotted. Note the lack of the backlash artifact. In summary, over these very small motion ranges, there appears to be a difference between the intended and achieved position of about 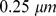 (compare desired and achieved position in backlash measurement Figures), which is roughly the magnitude of the backlash.

Whilst backlash is only about 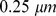, we also explored whether there might be additional forms of hysteresis over larger bidirectional motions. This is possible as the shaft is slightly elastic, which might create peculiar non-linearities not present in a conventional geared system. We tested this by measuring bidirectional positioning accuracy over larger amplitude motions. Figure 10D1 to D4 shows the end points of 50 bidirectional motion cycles of different amplitudes, ranging from 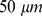 to 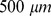. In other words, the objective was cycled between 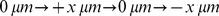, and we measured return accuracy at the 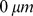 position. We implemented backlash correction (as described above) for these motions, but similar results are obtained without this correction. Over these larger motions there is excellent repeatability over time, as evidenced by the very tight clustering of the data points, which does not degrade at larger motion amplitudes. The outliers in Fig 10D3 are the product of the first two motions only. Bidirectional accuracy is well within 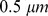 of the intended position (zero), with positions from the two motion directions separated by about 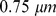. These values are substantially larger than the backlash, which argues that an extra form of hysteresis is present in these larger amplitude motions. We have not yet identified the source of this, but non-radial motion of the shaft is a possibility (the shaft swings slightly from side to side following direction changes). Importantly, increasingly larger step sizes do not produce increasingly larger errors, which indicates that the drive system is reliable over larger motions. If errors of this amplitude are unacceptable, then always approaching the desired location from the same direction (unidirectional motion) is the obvious solution (e.g. Fig. 8A). Finally, it may be possible to reduce the bidirectional error by using shafts with higher torque ratings, by passing the shaft through one or more fixed guide rings, or by correcting for the error in software. We have not as yet explored these options as the drive system is currently acceptable for our purposes. However, we have noticed that bidirectional repeatability errors increase greatly (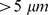) if the couplers securing the flexible shaft work loose because the setscrew loosens. One way of avoiding this is to paint a *small* quantity of Loctite on the setscrews using a toothpick.

## Discussion

We have presented a viable, low-cost, micro-positioning system for microscope stages. The accuracy of the system is as follows:

1. Repeatability in X and Y is under 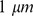.
2. Repeatability and motion linearity in Z is under 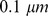.
3. Bidirectional positioning accuracy in Z 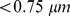.
4. The dominant source of Z positioning error over longer periods is thermal expansion and contraction of the microscope body itself.

These values are acceptable for the majority of multi-photon imaging tasks. We achieved this accuracy using very cheap parts and we believe there is sufficient flexibility in the design that it could be adapted to various different situations. We devote the Discussion to suggesting ways in which the system could potentially be improved.

### Decreasing slow drift

The largest errors we found originated not from our positioning system but from thermal expansion and contraction of the microscope body itself. The magnitude of this drift can be reduced if needed by redesigning the microscope and stage. Firstly, lowering the objective height will decrease the thermal sensitivity proportionately. Secondly, a major contributor of the expansion is due to the fact that the microscope gantry is aluminum but the sub-stage is supported by steel posts. The lower thermal expansion coefficient of the steel means that it can't keep pace with thermal expansion of the gantry. In theory, replacing the steel posts with aluminum would decrease the thermal sensitivity of our microscope from the current 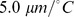 to about 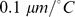.

### Improvements to fine positioning

We are using stepper motors with a full step size of 0.9° (400 steps/revolution). Microstepping improves resolution at the cost of unequal step sizes. Using motors with finer full-step sizes would help to combat this issue. Vexta's 1000 steps/revolution motors (such as the PK546PMB from OrientalMotor.com) driven at 1/4 or 1/8 steps would likely provide improved linearity and positioning accuracy. This is achieved not only by the smaller full step size, but also by the 5-phase design, which the manufacturer claims will eliminate microstepping positioning errors (we have not verified this). However, switching to these motors will likely require Vexta's custom 5-phase driver ($200 per axis) and also modification of the microcontroller code. Whilst this may be a valuable upgrade for Z-positioning, it is unlikely to be required for X and Y. Another alternative is the use of geared stepper motors. Whilst these are potentially more expensive, they can provide both high torque and very fine step sizes. However, geared stepper motors can introduce backlash and may have lower maximum speeds than non-geared units. A variety of geared Vexta stepper motors are available at *OrientalMotor.com* and cheaper Nema motors at www.stepperonline.com. Finally, substituting the 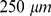 per revolution Newport HR-13 micrometer for the bulkier HR-1 (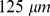 per rev.) should halve the RMS positioning error (Fig. 7D3) at the cost of halving linear speed of the stage.

### Improving absolute positioning

Although the flexible shafts have various advantages, they may be an important source of non-linearities which impact absolute positioning accuracy. Accuracy may be improved by exploring different shaft options: shorter shafts, higher torque shafts, or passing the shaft through guide rings. Utilizing a different mechanical coupler is another possibility. A variety of couplers are available at *SDP-SI.com*, with spline-based couplers being a obvious possibility if the user wishes to use micrometer-actuated linear translators. Alternatively, different gear mechanisms such as rack and pinion (e.g. the Olympus focusing mechanism) or lead-screw based translators (e.g. ThorLabs DT25) would provide a more direct way of coupling stepper rotation to linear motion. High precision lead screws are also available (e.g. www.universal-thread.com). These approaches may provide an improvement in bidirectional repeatability. We did not explore this option ourselves because we had micrometers and linear translators readily available at the start of the project, and because mounting the motors on our rig was easier with flexible shafts.

Our system runs in open-loop, without positioning information returning to the controller. Closed loop control might allow higher accuracy with existing hardware. Whilst Vexta do sell stepper motors with encoders, the encoder resolution is equal to the motor's full step size and so is not sufficient to encode micro-steps. High resolution (10,000 step) encoders are available from the amateur astronomy community (e.g. www.webstertelescopes.com/TELESCOPE_ENCODERS.htm), and could readily be incorporated into our design. In the event that we later wish to add encoders, the motors on our rig are all dual-shaft.

### Controller upgrades

The main drawback of our existing controller software is that it is currently unable to produce step pulse rates in excess of 4.3 kHz. This restriction places an artificial upper limit on motion speed (Table 2), particularly if one wishes to drive stepper motors rapidly at high resolution. Thus, to make effective use of motors with finer step sizes our controller needs to produce higher pulse rates (up to about 30 kHz per channel should suffice for most purposes). In future it should be possible to achieve this value by making better use of the Arduino's hardware timers and counters (see *github.com/grbl/grbl* for a relevant project from the hobby CNC world).

### Construction challenges

With the instructions provided here it should be straightforward for other researchers to replicate our stage and obtain comparable motion accuracy. We believe we have solved most of the time-consuming design problems: Obtaining reliable, orthogonal, motions in X and Y was difficult as the stage had to be disassembled in order to test different configurations of translators and roller bearings. Our large and heavy (17 kg) stage ultimately required 4 pairs of translators with the driver pair near the center of gravity of the breadboard to achieve high motion repeatability. Smaller and lighter stages may well require less effort to set up and may even work well with roller bearings. Additionally, we were surprised to discover that thermal expansion had such a strong effect. We initially thought that the drift we observed was due to cumulative positioning errors originating from the drive system. The clue that this was not the case was that the drift was very gradual and did not resemble skipped steps.

Backlash turned out to be a fairly minor concern. Over small motion ranges it is only about 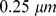 (Figure 10A–C2). Although we confirmed that backlash correction is possible, we generally run our stage with no correction, preferring to use unidirectional motions for cases where high accuracy is required. One reason for this is that hysteresis appears slightly greater for larger motion steps (Figure 10D) compared to very small steps (e.g. Figure 10A3 & B3), and since we have not explored this hysteresis in detail we cannot provide accurate correction values.

## Materials and Methods

### Imaging

All imaging was performed on a custom 2-photon microscope built by the authors. Coherent light was provided by a Chameleon Ti-Sapphire laser (Chameleon XR, Coherent Inc.) tuned to 920 nm. All images were acquired with an Olympus water immersion objective (XLUMPlanFLNW 20x, NA 1.0). Collected green light passed through a 750 nm high-pass filter (Semrock, FF01-750/SP-50), a FF552-Di02-50 dichroic (Semrock), and finally through a “green” band-pass filter (Semrock, FF01-525/50). Signals were detected with an R10699 photomultiplier tube (Hamamatsu) and amplified with an SR570 amplifier (Stanford Research Systems). Scanning was conducted using 3 mm Cambridge Technologies galvanometric scan mirrors (part number 8315 KB) and laser power regulated by a Pockels cell (Conoptics). The microscope was controlled by an unmodified version of ScanImage 3.8 [9]. Data acquisition and galvo control were performed using National Instruments PCI-6110 and PCIe-6343 boards.

The test slide was made by painting a green patch on a standard slide using a Sharpie accent highlighter. We used the darker, rather than paler green, pen. A cover slip was placed over the patch and it was sealed with clear nail polish. Pollen grains were imaged from a “mixed pollen grain slide” (*carolina.com*).

### Stepper motors and coupling hardware

X/Y motion was performed by Vexta PK243M-02BA stepper motors (OrientalMotor.com). For Z motion we chose a Vexta PK243M-01BA, since this motor is capable of achieving more revolutions per minute whilst maintaining reasonable torque (see also Table 1). This was important since the gear ratio in Z is double that of X/Y. In principle, there is no reason why the faster PK243M-01BA can not be used on all three axes. We also tried a cheap 1.8° Mercury motor (Sparkfun, ROB-09238) and found it to perform remarkably well for the price; indeed, we initially used one of these motors to drive objective motion when protyping the stepper motor and flexible coupler concept. The Vexta PK243M motors provide slightly better linearity and a smaller full step size compared to the Mercury motor. Finally, we experimented with a Vexta PK266-01A 1.8° motor, and found it generated too much vibration and was too slow for our application.

Power is transmitted to the micrometers via flexible shafts sourced from Stock Drive Products/Sterling Instruments (sdp-si.com). We selected from their catalog of imperial “remote control” shafts without a casing. In Z we used a 10′′ shaft with a torque rating of 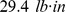 (A-7C12-10633). In X and Y we used 12′′ shafts with a torque rating of 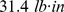 (A 7C12-12633), although lower torque shafts, such as the 8′′, 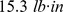, A 7C12-08533, also worked well. We also tried an 8′′ shaft with a torque rating of 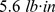 (A 7C12-08433), but we found this produced about 4° of backlash (which corresponds to about 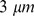 with a 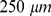/rev micrometer). Commercial microscopes usually have a rack and pinion gear for moving the objective; the fine focus knob can therefore be directly coupled to a stepper motor without the flexible drive shafts used here. Short flexible couplers are still required, however, to cope with inevitable minor misalignment errors between the motor spindle and fine focus knob.

We machined cylindrical male/female adapters for both ends of the flexible shafts: to couple shafts to the micrometer thimbles and to the motors (see Fig. 2). We used a coupler for the flexible shaft-to-motor connection because the motor spindles are 5 mm in diameter whereas the flexible shafts are designed for 1/4′′. We did try running our drive system without this coupler and no obvious problems occurred, but doing this mis-aligns the axes of the flexible shaft and motor spindle. Users not wishing to machine components could couple the shaft directly to the motor spindle and use glue or tubing and tube-couplers to connect the other side of the flexible shaft to the micrometer thimble.

### Choosing a stepper motor

We suggest purchasing a Vexta stepper motor from *OrientalMotor.com*, as they have a wide selection. First choose a combination of step size and micrometer gear ratio that will provide the a suitable balance between minimum step size and maximum speed. We provide Table 2 to help make the decision. Note that the maximum speed that the micrometers will tolerate is unknown. For reference, we have successfully driven our Z axis to speeds of 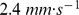 (using full steps), which corresponds to about 590 RPM. The Vexta 1.8° and 0.9° motors are standard stepper motors and can be controlled with cheap driver boards (as discussed below). The Vexta 0.72° and 0.36° motors will require more expensive driver boards from *OrientalMotor.com*. Also note that the 1.8° and 0.9° motors produce equal step sizes only at full and half steps (Fig. 7). The 0.72° and 0.36° motors are claimed to be linear across all micro-step sizes.

Once you have chosen a motor full step size, examine the speed/torque curves of motors in that class and choose one that produces at least 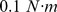 at the maximum speed you wish to reach. In X and Y we begin to see stalling when the motor reaches speeds that correspond to a torque less than about 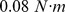. In Z, however, we continue to see stall-free motion down to at least 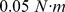. Likely this is due to the gear ratio being lower in Z than in X and Y. In practice, for absolute positioning tasks, we use 1/2 steps (minimum step sizes of 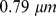 in X and Y and 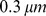 in Z) with maximum speeds of 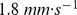 in X and Y and 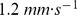 in Z. We use this combination as it produces a good balance between speed and positioning accuracy.

The user should edit the global variables at the top of the *OpenStage.ino* source file in order to select suitable step size and speed limits so that the motors do not stall or skip steps. Stalling is obvious, since the motor will stop rotating. Large numbers of skipped steps are also obvious: the stepper motor will briefly produce a characteristic grinding-like noise and the drive system will then lose position by a few tens or hundreds of microns. Motors may skip steps or stall for a variety of reasons, and this is most likely to occur during fast motion and under the following circumstances:

1. Motors driven beyond their torque limits (usually due to driving them too fast) will stall.
2. Not providing sufficient current for higher speed motion will lead to a stall. However, we have found there to be a current “sweet spot” at which a motor performs best. Providing too much current can lower the maximum attainable speed. To find the optimum current we observed motor motion whilst adjusting the current limit potentiometers on the driver board and monitoring current consumption using a DC power supply that provides this value on an LCD display.
3. Accelerating the motor too quickly or providing a choppy drive signal at high speeds will cause stalls and/or skipped steps.
4. Due to the way the magnetic field creates torque, stepper motors constitute a mass-spring system and are prone to resonances. These resonances can lead to skipped steps or stalls and occur in what is known as the “mid-band instability”, which is a narrow region in the middle to high end of the torque/speed curve. Accelerating harmlessly through the instability is often possible, but operating within it is not possible. Since resonances can be damped, a suitable damping device will resolve this issue. Stepper motor dampers consist of a plastic disk (which may be filled with viscous gel) that is attached to the free shaft of a dual-shaft motor. If the mid-band instability becomes a problem then a damper may be called for. Damping devices can be “DIY” items, such as a roller blade wheel, or a specially designed item, such as a “Clean Damper” from OrientalMotor.com. In our system we noticed the instability sometimes during medium to fast (Speed Mode 4) analog-stick motions in Z when coarsely guiding the objective to the sample.

Although there may appear to be many sources of skipped steps and stalls, with correctly driven motors, stalls will never occur in practice and isolated skipped steps will be very rare indeed.

### Electronics

Stepper motors can be driven with any chopper microstepping driver. Stepper driver boards distribute power to the motors and take care of the operations required to implement micro-stepping. TTL logic inputs set step size, rotation direction, and motor power state. The stepper motor executes a step of the desired magnitude and direction each time a TTL pulse arrives at it the Quadstepper's “step” input. Any programmable device capable of producing TTL pulses can be used to issue control signals. The pulse rate of the step input determines speed of rotation.

We used a 4-channel “Quadstepper” motor driver board (Sparkfun, ROB-10507). A single-channel version is also available (Big Easy Driver, Sparkfun, ROB-11699). Alternative driver boards can be sourced from Pololu Robotics & Electronics (www.pololu.com). Cheap enclosed driver boards by Nema are available from www.stepperonline.com. Finally, more expensive Vexta driver boards are available from *OrientalMotor.com*. Note that changing the driver board to something other than the SparkFun units will likely require minor changes to the OpenStage source code.

The Quadstepper board is capable of driving motors with substantially higher power demands than those used on our rig. Driver current output is regulated by a small potentiometer on the driver board. It is important to set this potentiometer to the lowest value that produces smooth, stall-free, motions. Higher current settings will cause the motors to run unnecessarily hot and the driver IC chip itself can get very hot indeed. Our motors can be run with the driver board being only slightly warm to the touch. No heat-sinks are necessary. The motors we have chosen are a six lead design but the driver boards only require two pairs of leads. Switching the lead pairs switches the default motor direction.

Our controller is built around an Arduino Mega 2560 R3 (Sparkfun DEV-11061). A Sony PS3 DualShock 3 controller provides the input device and is connected to a USB Host Shield (Sparkfun, DEV-09947), which has drivers for other input devices such as the Nintendo Wii remote or the XBox 360 controller (*github.com/felis/USB_Host_Shield_2.0*). The USB Host Shield does not have drivers for older or newer versions of the Sony DualShock controller. Note that the USB Host Shield is not compatible with the ARM-based Arduino Due or the PIC-based ChipKIT boards. Stage position is displayed on a 20×4 character LCD display (Sparkfun, LCD-0025), which is based around the standard Hitachi HD44780 LCD controller. We used an RS232 Shifter (Sparkfun, PRT-00449) to interface between the Arduino and the PC's serial port. Whilst it's also possible to use the USB programming port for this purpose, having an extra serial port is more convenient for software development.

We used a 9 V DC power supply for the Arduino (Meanwell ELN-30-9) and a 24 V DC power supply for the stepper driver board (Meanwell CLG-100-24), although a single 24 V supply and a suitable voltage regulator for the Arduino would also work. The 24 V power supply we chose is a 4A unit capable of driving motors substantially more powerful than those used here. Parts were mounted in a metal case using stand-offs fashioned from nylon nuts and screws. Motors were connected to the case using DB9 serial cables and connectors. USB connections to the front of the case were provided using panel-mount cables (such as #908 and #907 from www.adafruit.com). We mounted red 5 mm LEDs to the front of the case using holders (Sparkfun COM-11147) to indicate when each axis is moving. The front of the case also has separate switches for each power supply. A complete components listing for our controller is provided in Table 5.

**Table 5:** Parts list for controller unit.

Item	Supplier	Part No.	Price ($)
Arduino Mega 2560	Sparkfun	DEV-11061	59
Quadstepper motor driver board	Sparkfun	ROB-10507	65
USB Host Shield	Sparkfun	DEV-09947	25
20×4 LCD display	Sparkfun	LCD-00256	18
RS232 Shifter	Sparkfun	PRT-00449	14
2-pin screw terminals	Sparkfun	PRT-08084	4
2x break away headers	Sparkfun	PRT-00116	3
2x female headers	Sparkfun	PRT-00115	3
4×5 mm LED holder	Sparkfun	COM-11147	2
2x Female DB9 connector	Sparkfun	PRT-00110	2
2×10 k trim pot	Sparkfun	COM-09806	2
2x rocker switch	Sparkfun	COM-10727	1
Adafruit Perma-Proto 1/4 size	Adafruit	ID:589	9
Female/Male jumper wires	Adafruit	ID:825	7
Breadboarding wire bundle	Adafruit	ID:153	6
2x terminal block	Adafruit	ID:677	5
USB A male to A female panel mount	Adafruit	ID:908	4
USB B male to B female panel mount	Adafruit	ID:907	4
USB cable - A/MiniB - 3ft	Adafruit	ID:260	4
Tiny breadboard	Adafruit	ID:65	4
Black hook-up wire	Adafruit	ID:290	3
Red hook-up wire	Adafruit	ID:288	3
Piezo Buzzer (PS1240)	Adafruit	ID:160	2
Meanwell 24 V PSU CLG-100-24	Mouser	709-CLG100-24	72
Meanwell 9 V PSU ELN-30-9^*^	Mouser	709-ELN30-9	30
200×4-40 nylon hex nuts	Mouser	561-G440	38
100×4-40 nylon screws	Mouser	534-9329	9
AC power entry module	Mouser	693-6220.2300	9
2×10ft DB9 M/M Cable	cablestogo.com	09449	20
PS3 DualShock	Amazon.com	B0015AARJI	38
Microtivity IL081 5 mm Assorted LED w/Resistors	Amazon.com	B004JO2PVA	6

This table lists all parts required to construct our controller. In addition to the above parts, the user will need to source or build a suitable case. We recycled an old case from a previous project. Many of these parts can be sourced from other suppliers, we simply list convenient US suppliers. Prices are rounded to the nearest USD and correct at the time of publication. Total cost of the above components is $471. ^*^A single 24 V 2 A power supply and a 9 V regulator would, of course, also work and cost less.

Note that for the USB host shield to work, the reset line may have to be jumpered to pin 7 on the shield. In addition, the USB host shield must be connected to the Mega's Serial Peripheral Interface (SPI) pins. We suggest not stacking the boards, as this blocks the use of some pins on the Mega (see Fig. 5A). The following connections must be made to the SPI pins: Mega 52 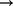 13 on shield; Mega 50 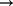 12 on shield; Mega 51 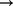 11 on shield; Mega 53 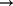 10 on shield; Mega 53 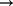 10 on Mega. The reset lines on the Mega and the shield must also be connected.

### Serial communications

Serial commands to control the stage from a PC go via the Serial1 connections on the Arduino Mega. These are connected to an RS232 shifter (Sparkfun) to convert the signals to the higher voltages required for PC serial communication. The serial shifter is powered by the +3.3 V line from the Arduino. The terminator character (‘$’) is only transmitted on commands which require it. The input buffer on the OpenStage is flushed following each command. Commands are sent and data received as plain ASCII, not binary. The commands for initiating and controlling motions via the serial port are described in Table 3. The parameters described in this table also affect motions triggered by double-clicking one of the right hand (stored location) buttons on the DualShock. Commands not related to automated stage motions are described in Table 4.

### Suggestions for building your own system

Building the controller unit is simple because only three boards are strictly necessary: Arduino Mega, USB Host Shield, and a Quadstepper driver board. Optionally, one can add an LCD display and a Serial Shifter for communicating with a PC using a second serial interface. All Arduino pin connections are prominently described in the source code and some are also shown in Fig. 5A. Read the comments in the source code before wiring the system. The Quadstepper board motor outputs go to DB9 female sockets attached to the case. We purchased M/M serial cables, cut them in half, connected one end to the female DB9 socket on the case and the other end is soldered to the flying leads on the stepper motor.

We recommend initially testing the system with the motors disconnected from the stage hardware. Attach a piece of tape to the spindle and ensure that the motor rotates as expected in response to commands from the DualShock and, optionally, the PC. For testing, it may be helpful to have a spare Arduino Uno, a spare USB Host Shield, and a spare stepper driver board (possibly a single channel Big Easy Driver from Sparkfun). Also useful for testing are an oscilloscope, laboratory power supply (such as a Madell TPR3003-3C), and a multimeter. Commanded motor speed can be confirmed by feeding the step signal into an oscilloscope and measuring the pulse frequency.

## Supporting Information

Movie S1 (MOV)

Movie S2 (MOV)

Movie S3 (MOV)

## References

[1] Denk W, Strickler J, Webb W (1990) Two-photon laser scanning fluorescence microscopy. Science 248: 73–6. 2321027 10.1126/science.2321027

[2] Denk W, Svoboda K (1997) Photon Upmanship: Why Multiphoton Imaging Is More than a Gimmick. Neuron 18: 351–357. 9115730 10.1016/s0896-6273(00)81237-4

[3] Denk W, Yuste R, Svoboda K, Tank D (1996) Imaging calcium dynamics in dendritic spines. Cur Op Neurobiol 6: 372–378. 10.1016/s0959-4388(96)80122-x 8794079

[4] Svoboda K, Denk W, Kleinfeld D, Tank D (1996) In vivo dendritic calcium dynamics in neocortical pyramidal neurons. Nature 385: 161–165. 10.1038/385161a0 8990119

[5] Tsai P, Friedman B, Ifarraguerri A, Thompson B, Lev-Ram V, et al (2003) All-optical histology using ultrashort laser pulses. Neuron 39: 27–41. 12848930 10.1016/s0896-6273(03)00370-2

[6] Ragan T, Kadiri L, Venkataraju K, Bahlmann K, Sutin J, et al (2012) Serial two-photon tomog- raphy for automated ex vivo mouse brain imaging. Nat Meth 9: 255–8. 10.1038/nmeth.1854 PMC3297424 22245809

[7] Mainen ZF, Maletic-Savatic M, Shi SH, Hayashi Y, Malinow R, et al (1999) Two-Photon Imaging in Living Brain Slices. Methods 18: 231–239. 10356355 10.1006/meth.1999.0776

[8] Nguyen QT, Tsai PS, Kleinfeld D (2006) MPScope: a versatile software suite for multiphoton microscopy. J Neurosci Methods 156: 351–9. 16621010 10.1016/j.jneumeth.2006.03.001

[9] Pologruto TA, Sabatini BL, Svoboda K (2003) ScanImage: flexible software for operating laser scanning microscopes. Biomed Eng Online 6: 2–13. 10.1186/1475-925X-2-13 PMC161784 12801419

[10] Guizar-Sicairos M, Thurman S, Fienup J (2008) Efficient subpixel image registration algorithms. vOptics Letters 33: 156–158. 10.1364/ol.33.000156 18197224

[11] Campbell R, Honegger K, Qin H, Li W, Demir E, et al (2013) Imaging a Population Code for Odor Identity in the Drosophila Mushroom Body. J Neurosci 33: 10568–81. 23785169 10.1523/JNEUROSCI.0682-12.2013 PMC3685844

